# What is beyond the edges? Gated communities and their role in China’s desire for harmonious cities

**DOI:** 10.1186/s40410-020-00122-x

**Published:** 2020-10-19

**Authors:** Badiaa Hamama, Jian Liu

**Affiliations:** 1 School of Architecture, Department of Urban and Rural Planning, Tsinghua University, Beijing, China; 2 School of Architecture, Tsinghua University, Beijing, China

**Keywords:** Chinese gated community, Qualitative urbanism, Human-oriented approaches, Openness, Gatedness as opportunity

## Abstract

During the rapid process of urbanization in post-reform China, cities assumed the role of a catalyst for economic growth and quantitative construction. In this context, territorially bounded and well delimited urban cells, globally known as ‘gated communities’, *xiaoqu*, continued to define the very essence of Chinese cities becoming the most attractive urban form for city planners, real estate developers, and citizens alike. Considering the guidelines in China’s National New Urbanization Plan (2014–2020), focusing on the promotion of humanistic and harmonious cities, in addition to the directive of 2016 by China’s Central Urban Work Conference to open up the gates and ban the construction of new enclosed residential compounds, this paper raises the following questions: As the matrix of the Chinese urban fabric, what would be the role of the gated communities in China’s desire for a human-qualitative urbanism? And How to rethink the gated communities to meet the new urban challenges? Seeking alternative perspectives, this paper looks at the gated communities beyond the apparent limits they seem to represent, considering them not simply as the ‘cancer’ of Chinese cities, rather the container of the primary ingredients to reshape the urban fabric dominated by the gate.

## Introduction and methodology

Since the opening up and economic reforms in the late 1970s, China’s urbanization has been undergoing an unprecedented rapid process. From the late 1980s to 2015, China’s urban population increased from roughly 20–56%, and the number of cities increased from 193 to 658 (China’s ‘*National New Urbanization Plan* 2014–2020’). Given the serious challenges resulted from the unsustainable urbanization path, China introduced several urban experiments such as eco-cities, low-carbon cities, and sponge cities to face the growing critical urban issues. Most of these guidelines were often criticized for being too general and less specific, leading to confusion and misinterpretations (Liu et al. 2014). In fact, most of the adopted approaches were quantitatively-oriented, resulting in many contradictions and problems, such as the difficult task of integration of the migrants into urban society, inefficient and extensive construction land, unreasonable spatial arrangement, population pressure in the main urban areas of mega-cites, traffic congestion, pollution, declining urban culture, environmental deterioration, etc. (China’s ‘*National New Urbanization Plan* 2014–2020’).

Today, after decades of rapid urbanization, China is entering a decisive stage for building a well-off society, as part of a general plan for economic transformation and upgrading, boosting the process of socialist modernization and realization of the Chinese dream of ‘great rejuvenation’ of the nation. Compared to the fast and quantitative urbanization, which characterized the previous decades, China is shifting now its urban agenda towards ‘qualitative-oriented’ approaches. The need to overcome the traditional extensive urbanization strategy is clearly reflected in China’s *National New Urbanization Plan* (2014–2020). The aim is to promote an urban development model socially inclusive, economically practical, physically compact, ecologically resilient, and able to sustain a pluralistic and open modern culture. In the new urban agenda, mainly five key areas have been identified for qualitative urban development: ‘optimization of the urban spatial structure’, promotion of ‘people-oriented development models’, ‘upgrading of the urban economy’, building ‘green and cultural cities’, and ‘modernization of urban governance’. To achieve qualitative urbanization, four initiatives have been highlighted: ‘creative city’, ‘compact city’, ‘green and cultural city’, and ‘smart city’. There is a clear urge to plan the layout of urban spatial functions as a whole, and assure the proper mixing of urban land use functions; promote the integration of economic and social planning, urban planning and land use planning.

However, when talking about urban renewal and the promotion of harmonious cities in China, the optimization of their spatial structure, and the promotion of people-oriented approaches, it’s paramount to understand which mechanisms and approaches will be adopted to integrate the gated communities, as one of the basic entities and most visible features of these cities at the neighborhood level. In a symbolic period in which China is looking to prevent and control ‘urban diseases’ and encourage the building of harmonious, livable, and vibrant cities, it is important to reflect on what would be the role of the gated communities in China’s desire for the achievement of human-qualitative urbanism, and how will they evolve along with the new urbanization agenda. Examining the existing studies on gated communities in China, and adopting a human-oriented approach, this paper attempts to give possible answers to the above mentioned questions, discuss the challenges and opportunities facing the opening up of the gated communities in China.

To give the reader a comprehensive understanding of the topic, the paper is designed as follows. The first section is a general literature review of the phenomenon of gatedness from the global perspective, and its specific position in the Chinese urban context. The second section critically summarizes the most important debates on the issue of gated communities in China. The third section discusses how to rethink the gated communities at the physical and grassroots level. The last section provides conclusions of the main findings and suggestions for future research.

## Literature review: the gate at the global level and its specific position in the Chinese context since ancient times

### The gated community as a global phenomenon

Gated communities are generally defined as ‘*walled or fenced housing developments, to which public access is restricted, characterized by legal agreements which tie the residents to a common code of conduct* and usually collective responsibility for management’ (Atkinson and Blandly 2005). The phenomenon of gated communities is nowadays recognized as a global tendency. It has been analyzed from a multidisciplinary perspective including sociology, politics, economy, geography and urban design. Most of the previous research is focusing on the visual alleged negative effects of the gated community in terms of social segregation and exclusiveness, spatial fragmentation and division (Blakely and Synder 1997; Atkinson and Blandy 2005; Glasze 2005). However, various researchers emphasized the importance of analyzing the phenomenon based on the local forces and variables that drive or hinder the growth of a specific gated community market in different global contexts (Le Goix and Webster 2009; Freeman 2003; Thuillier 2005; Burke 2001; Lentz 2006; Glasze and Alkhayyal 2002). In the case of gated community in China, the emphasis is especially addressed to understand the practice of enclosure as a unique characteristic, which distinguished the Chinese city since ancient times (Wu 2005; Huang and Low 2008; Miao and Zhen 2009; Junxi 2014). In fact, enclosed residential developments in China, although sharing some similarities with those that can be found around the world, they remain a distinctive and very unique feature of the Chinese urban fabric (Webster et al. 2006; Le Goix and Webster 2009; Miao 2009).

### Walling and gating in the Chinese urban landscape: a distinct feature since ancient times

**Fig. 1:**
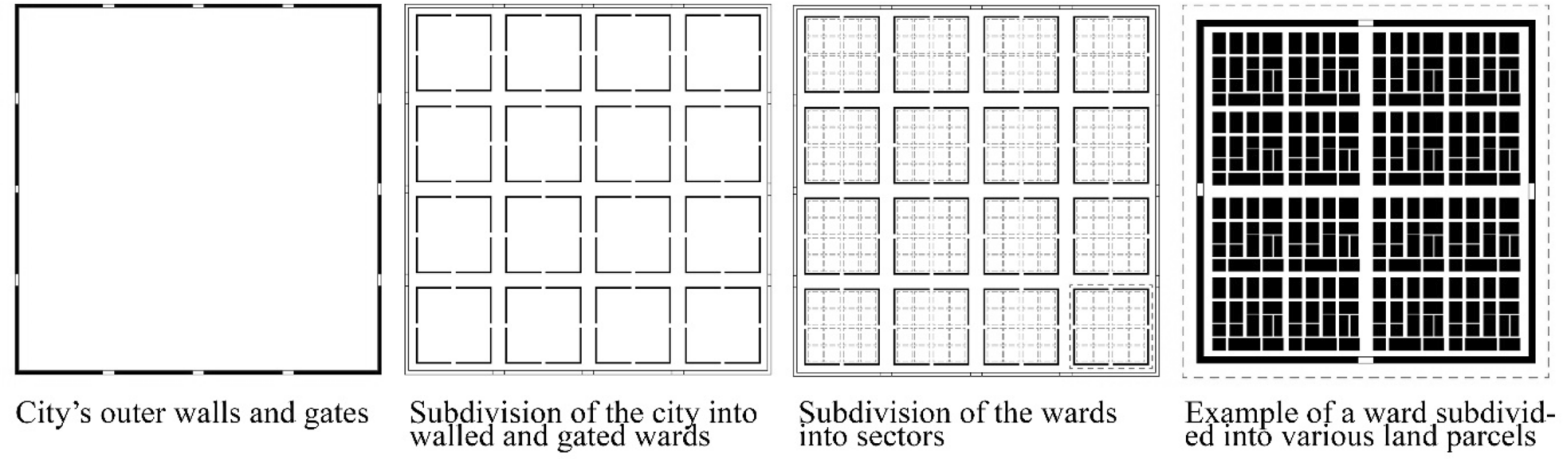
Schematic representation of the typical structure of a Chinese city during the Tang Dynasty (Source: Hamama 2017)

**Fig. 2:**
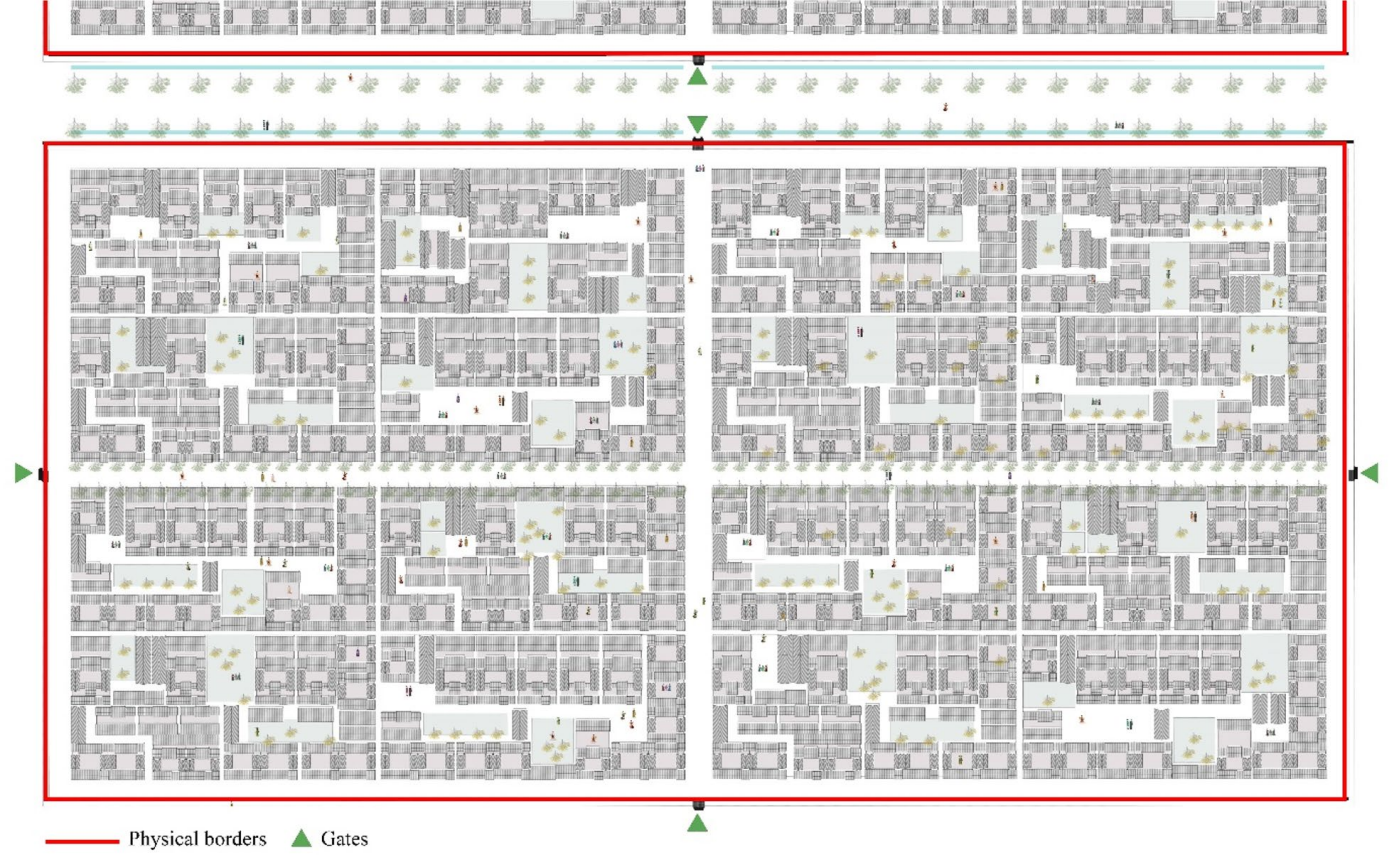
Axonometric view of a typical residential ward of sixteen sectors. Each residential ward, was walled and gated and subdivided into different sectors and land parcels through the internal road networks. Wards had different dimensions, the smallest one could range between 500 × 500 m and the largest one 1000 × 500 m (Source: Hamama 2017)

Gates and walls represented for long time a distinct feature of the Chinese urban landscape (Wu 2005; Huang 2006; Huang and Low 2008; Pow 2009; Liu 2019). Prior to the Song Dynasty (960–1279 AD), cities had external walls and internal walled-off wards (Fig. 1). Although the phenomenon of gated community is now recognized globally, the enclosed physical form of gated settlements—along with the collectivism oriented culture—is deeply embedded in Chinese history of city building and society, and it can be dated back to the walled cities of the pre-socialist centralised feudal monarchy (Fig. 2), the enclosed *danwei*^1^ of the socialist period, till the contemporary gated communities, which dominated the Chinese urban layout, especially, after the economic reforms since 1978.

The Chinese orientation towards life takes shape in an environment demarcated by walls as a device to create an intimate and distinct ambience. According to Miao and Zhen (2009), the enclosed open space embodies a very distinct difference in the building-space relationship of housing between the Western and Chinese traditions. Compared to the openness featured by the residential blocks in the West, Chinese tradition is more inclined to absorb and internalize the open space through the use of walls and buildings around it (*ibid.*).

### The re-emergence of neighborhood enclosure in China amidst housing and land marketization

The transition in the meaning of the gate since the decline of the *danwei* system (Wang 2013) can be embraced only if positioned in the broad transition from a planned economy to a market economy since the late 1970s. In fact, in the 1990s, the transformation of China from a welfare-based housing system and the establishment of a housing market represented a turning point in Chinese urban history. The new housing reforms allowed the market forces and the private enterprises to play an increasing role in the economy as a whole and in the production and consumption of housing in particular (Wang and Murie 1999). Giving also the new challenges imposed on the Chinese economy due to the Asian Financial Crisis, China identified the commercialization of housing provision as a new growth alternative that would stimulate domestic demand and drive future urban and economic development (*ibid.*).

The introduction of land reforms in the 1980s played a decisive role in the establishment of a housing market system in China. The quasi-nullified value allocated to land and to its location during the socialist period became the main driving force for economic growth and future urban development. In this period, ‘home’ became the most important new form of private property for urban Chinese (Feng 2003). The wall re-emerged embodying new meanings and reflecting the mentality of a society in gradual transition from socialist-collectivism to a market-oriented system (*ibid.*). Governments of all levels have included gating residential areas as part of their programs (Miao 2003), and most of the newly built housing during the process of housing commodification were surrounded by walls or fences (Wang and Murie 1999). The collapse of the *danwei*, with its rigid socio-spatial organization, the establishment of a consumerist society, and exclusive consumption, resulted in a crescent desire to escape the previous collectively monitored and organized social life (Hamama et al. 2019). In this context, the making of residential spaces became the main economic driver and enclosed residential developments represented the most privileged urban form favoured by local governments, real estate developers and citizens.

Yet, rather than the gate in itself, the new value associated to land and the housing commodification processes are the major factors which led to social segregation and physical fragmentation in post-reform China (*ibid*.). Pursuing the lucrative profits from land sales has become a top priority for local governments, which resulted in an unprecedented housing development boom in Chinese history (Deng et al. 2011; Wang 2013). However, while the economic reform has created a commercial housing development industry, it has not produced enough high-income and middle income families to sustain the emerging housing market (Wang and Murie 1999).

## Debates

### Contemporary gated communities in China between cultural continuation and neo-liberal urbanization

After the collapse of the work unit estates, and the introduction of housing and land market, enclosed neighbourhoods became a remarkable feature in the Chinese urban context (Bray 2005; Webster et al. 2006; Huang 2006; Miao and Zhen 2009; Junxi 2014; Zeng et al. 2016). Enclosed neighbourhoods in post-reform China acquired a strong presence to the point of being an ‘institutionalized’ practice (Wester et al. 2006; Huang and Low 2008; Zeng et al. 2016).

In the existing literature, there are many schools of thought explaining the reasons behind the prevalence of neighbourhood enclosure in post-reform China. One category of scholars (Dutton 1998; Huang 2006; Huang and Low 2008) sustain that, in spite of the profound political, economic and social changes, the popularity and continuity of enclosed urban settlements in the Chinese socialist and post-reform eras are supported by the persistence of the concept of collectivism-oriented culture deeply embedded in Chinese society. The collectivist culture, which is considered more important in Confucian tradition, is also one of the major points that differ China’s philosophy for the construction of gating cities from the Western culture (Huang and Low 2008). Although Huang and Low (2008) suggested that the discourse of fear can hardly be associated to the production of gated communities in China, Miao (2003) sustained instead that gated communities in China are based on the distinction between the different social classes, particularly rural migrants living in cities.

In the view of others, cultural continuation alone is not sufficient to explain the phenomenon of gated communities in China, which is instead the result of complex and radically different political, economic and social mechanisms that resulted in a ‘neo-liberal urbanization’ (Wu 2010). Junxi (2014) sustains that the main actors behind the large emergence of gated community in post-reform China are the local state, the private developers, and the wealthy urban residents. In 1982, the *Constitution of The People’s Republic of China* recognized the state as owner of urban land and the local governments as agent of urban land management, which has encouraged private estate developers to seek profit through large scale housing developments amidst a booming real estate market soon after the housing and land reforms (*ibid*). Gated communities became the main dominant development pattern in the commodity housing market, as it has been adapted to the complicated social, economic and environmental urban contexts throughout the country (Miao and Zhen 2009).

For other scholars, although framed within the existing traditions, the long lasting practice of gating in Chinese cities has been supported officially by the state as a tool for social control and preferred by the residents for its enclosed management and sense of security (Miao 2003; Webster et al. 2006; Zeng et al. 2016). The socialist state under a planned economy, used the gate as a tool to facilitate the delivery of the limited public services and help exert political control down to the grassroots, and the subsequent economic reforms and privatization of housing, made gating even more attractive to the government for continued control in an increasingly liberal society (Huang 2006). Miao (2003), stated that the primary reason for gating in China is security; during the transition from a planned economy to a market-oriented economy, the government has social stability and order as its topmost priorities, and gating represented a quick at hand tool to achieve that goal. Governments at all levels have been encouraged to include gated residential areas as part of their programmes, and gating became an important criterion in deciding if a community will be awarded the official title of ‘Civilized and Safe Residential Quarter’ (*ibid.*). According to Tomba (2010), in an increasingly stratified and complex Chinese society, ‘gating and privatization of residential spaces are acts of political classification, framed within existing traditions’, and used by the state as a tool to maintain order and social stability.

### Contemporary gated community as the undesired most-visible and undeniable urban feature of the Chinese urban fabric

**Fig. 3:**
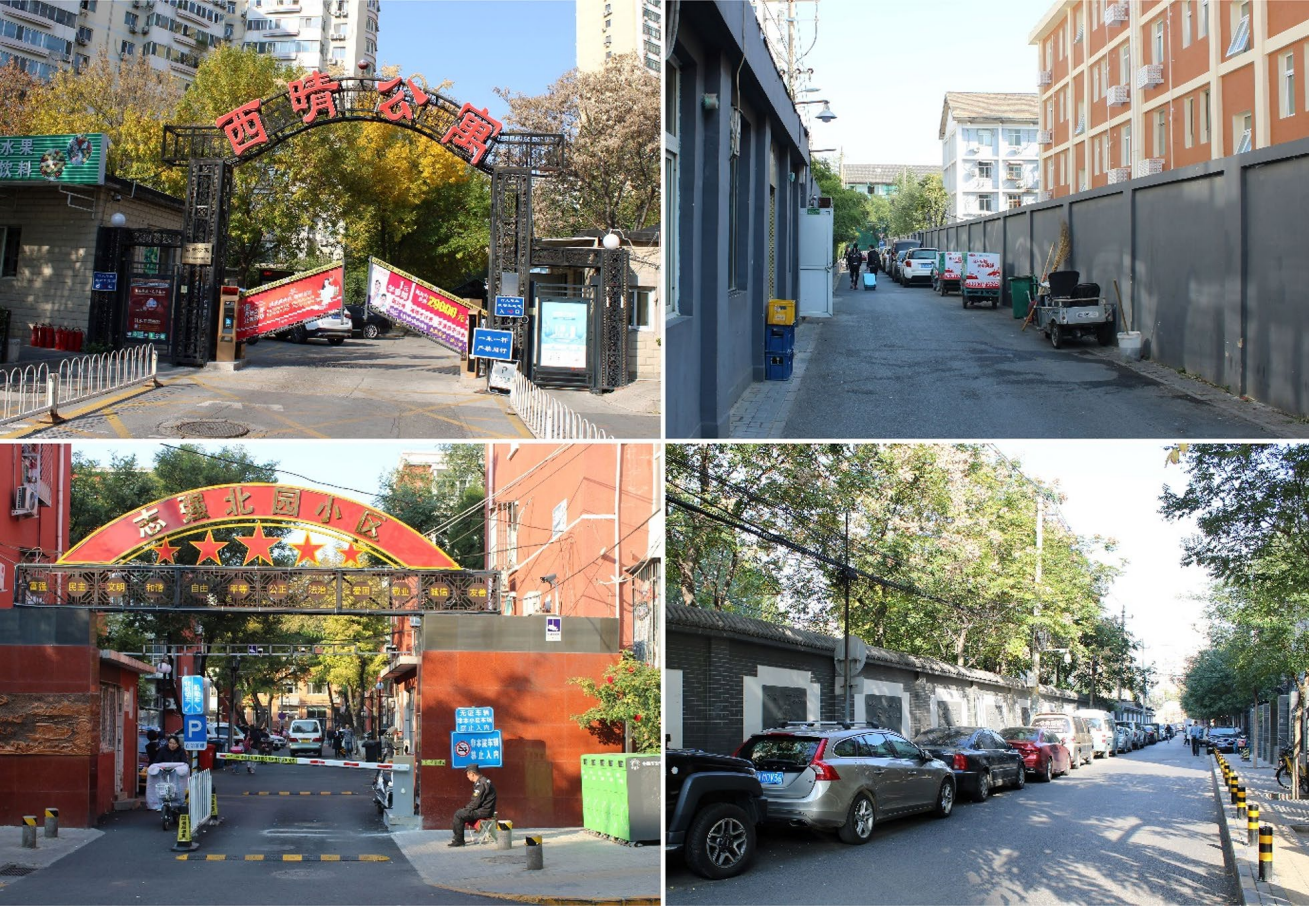
Gates and walls of some residential neighborhoods in Beijing (Photo Credit: author, 2019)

Often defined as the ‘cancer’ of Chinese cities, enclosed neighborhoods are one of the most evident and unquestionable features of the Chinese urban fabric since ancient times and till nowadays. Chinese cities, generally defined as ‘walled’ and ‘gated cities’ (Knapp 2000; Huang and Low 2008), have been continuously dominated by the prevalence of ‘inward-looking’ communities (Bray 2005; Huang 2006), and the attitude towards the gated communities seems not to be perceived as a social problem by both ordinary people and the decision makers (Junxi 2014). However, although its prevalence as the basic entity defining the form and structure of city space (Fig. 3), gated community phenomenon is becoming also the source of continuous debate among scholars, policy makers, and urban planners in China. The major debate, in the existing literature, is focusing on the mechanisms underlying the existence and persistence of the gated communities in the Chinese urban context, on how to achieve the ideal of an ‘open city’, and how to replace the existing urban form dominated by gated community types with a revolutionary urban model, with the hope to solve some of the urban issues facing Chinese cities (Junxi 2014; Zeng et al. 2016).

### Open-up the gates—the controversial directive of 2016

**Fig. 4:**
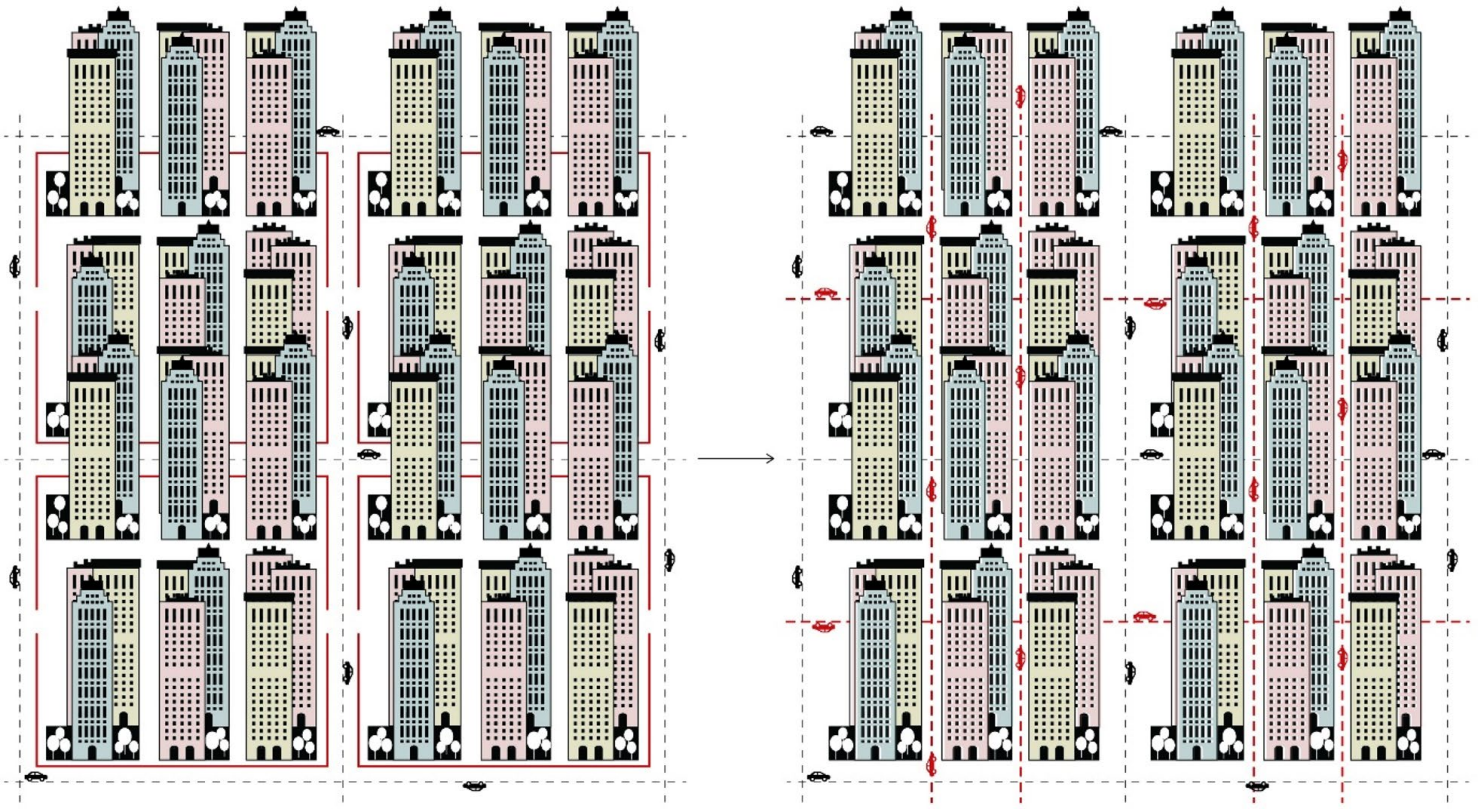
Open-up the gates: the aim of the new directive is to add a new layer of roads within the currently enclosed superblocks (Source: Hamama 2017)

During its historical meeting in 2016, China’s Urban Work Conference delivered a new set of urban guidelines urging for the creation of more livable, green and sustainable cities. Among these guidelines, one in particular targeted the gated communities: ‘*In principle, no more enclosed residential compounds will be built. And existing residential compounds will gradually have their interior streets integrated into public road network*’ (Fig. 4).

The proposal raised a strong debate and opposition from the public,^2^ as well as criticism from legal experts, who suggested that the policy contravenes the 2007 *Property Right Law of The Peoples*’* Republic of China*: ‘*roads and other public areas and facilities within a building zone are jointly owned by owners, with the exception of the public roads belonging to a city ownership*’. Both the *People’s Daily* and the Ministry of Housing and Urban–Rural Development have cited the prevalence of the street-block system in many developed economies to justify the proposal of banning the construction of more gated communities and open-up the existing ones. The policy, to date, has not been implemented, and its failure is another proof that the success of any new urban strategy or policy depends on the way the bottom up forces are involved in the transitional process of desirable transformations. Effectively, according to Wang et al. (2016) the opening up of the gated communities in China is still faced by a clear opposition and doubtful attitude within a large number of homeowners. Consequently, it is paramount, among others, to introduce new mechanisms to reinforce the involvement of the bottom-up forces for the creation of urban places with shared values that reflect the needs and interests of the people in first place.

### ‘Community Building’ and the limited participation at the neighborhood level

**Fig. 5:**
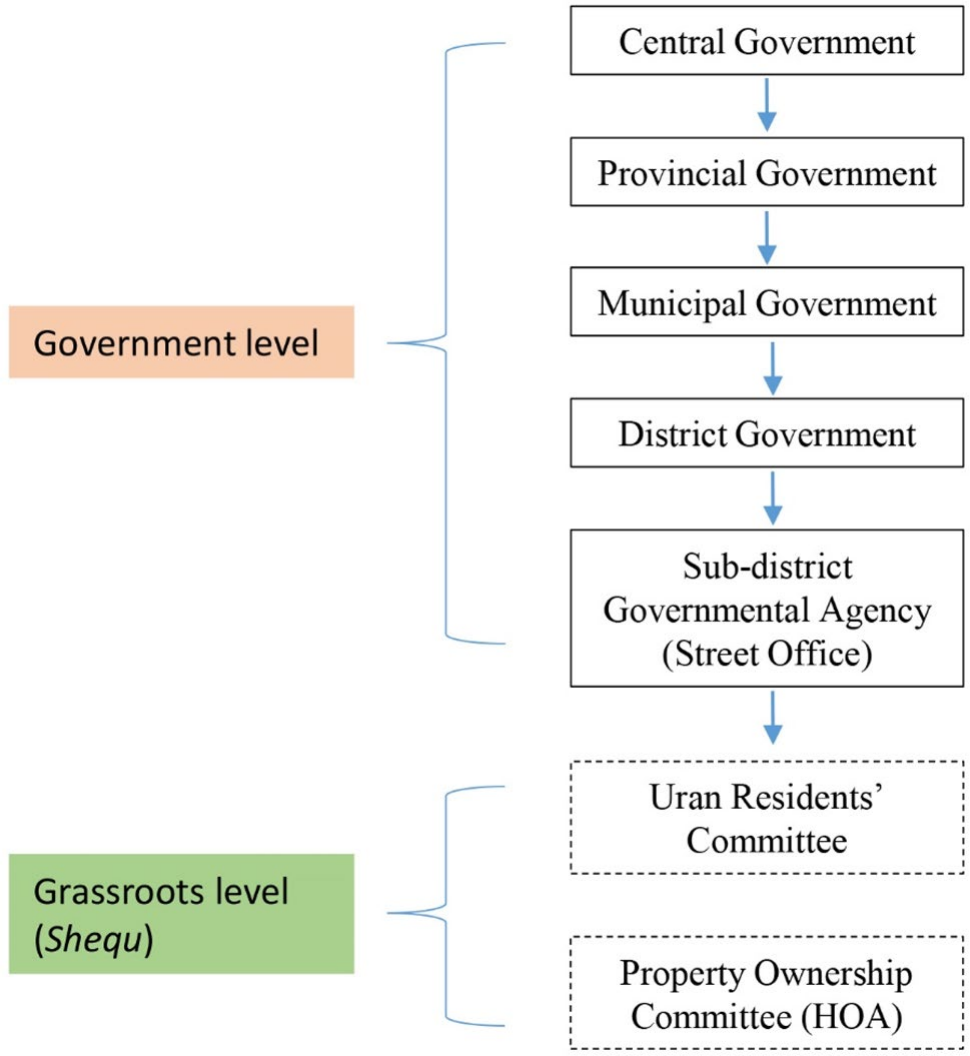
The hierarchical administrative structure in China (Source: by the author)

With the rapid urbanization in China, the local community system had been transformed in a considerable way. Since the dissolution of the *danwei* system of the socialist period, the concept of community*—shequ* was introduced in the 1990s (Xia 2008), as a way to cope with the new emerging socio-spatial challenges. In this transitional urban context, at the neighbourhood level, the Urban Residents’ Committee’—*Juweihui*, took over the welfare functions previously performed by the *danwei*, becoming the main tool of action at the local community level. In China, the administrative scheme is based on a hierarchical and multi-level system composed mainly of governments at the top, then district and sub-district governments, while the Urban Residents’ Committee and Property Ownership Committee, called also Homeowners’ Association, are theoretically independent organizations at the grassroots level (Fig. 5).

The concept of community building—*shequ Jianshe—*became a type of ‘urban governance’ (Junxi 2014). An officially recognized Urban Residents’ Committee was expected to be a ‘social collective’—*shehui gongtongti*—formed by people residing within a defined and bounded district (Huang and Low 2008). Bray (2005) and Xia (2008) sustain that the creation of *shequ* institutions are the result of ‘geographical zoning’ of social life, a manageable means for the ‘top-down’ creation of controlled citizens. Wu (2018) considers the residents’ committee as a ‘territorialized form of governance’, and extension of the State’s presence at the neighbourhood level, and consequently, largely different from the independent grassroots organizations in the West. In fact, although the Urban Residents’ Committee, is considered as an autonomous organization, it is largely dependent on the street office, especially at the financial level, and this resulted in disempowered residents with limited participation in neighbourhood affairs and management (Wu 2018; Zhang et al. 2018). However, in some commodity and enclosed housing developments, the Urban Residents’ Committee’s role has been partially overtaken by the Property Ownership Committee, or Homeowners’ Association, which seems to better reflect the residents’ interests, protecting their property rights, and at the same time reflecting their desire for more privacy (Wu 2018).

## Results and discussion

### Looking beyond the edges

The urban reality of Chinese cities is becoming more multifaceted and articulated, that it needs to be faced with alternative approaches more human-centred and less technically-oriented. The existence of the gated communities in today’s Chinese cities should be looked at beyond its physical borders, analysing instead how these could be exploited to achieve quality urban spaces addressed to enhance the well-being of the residents, rather than providing more road space to accommodate the increasing vehicular traffic. If the gated communities are criticized in the West for being a social problem, this may not be the same in China where people of different social classes and backgrounds lived accustomed to the enclosed urban settlements since ancient times and till nowadays (Zhang 2010). In fact, one of the main challenges facing the implementation of the opening-up policy is at the social level, specifically obtaining the consensus of the homeowners of the gated communities for the introduction of new infrastructures within their residential spaces, which are protected as mentioned above by the 2007 Property Right Law.

**Fig. 6:**
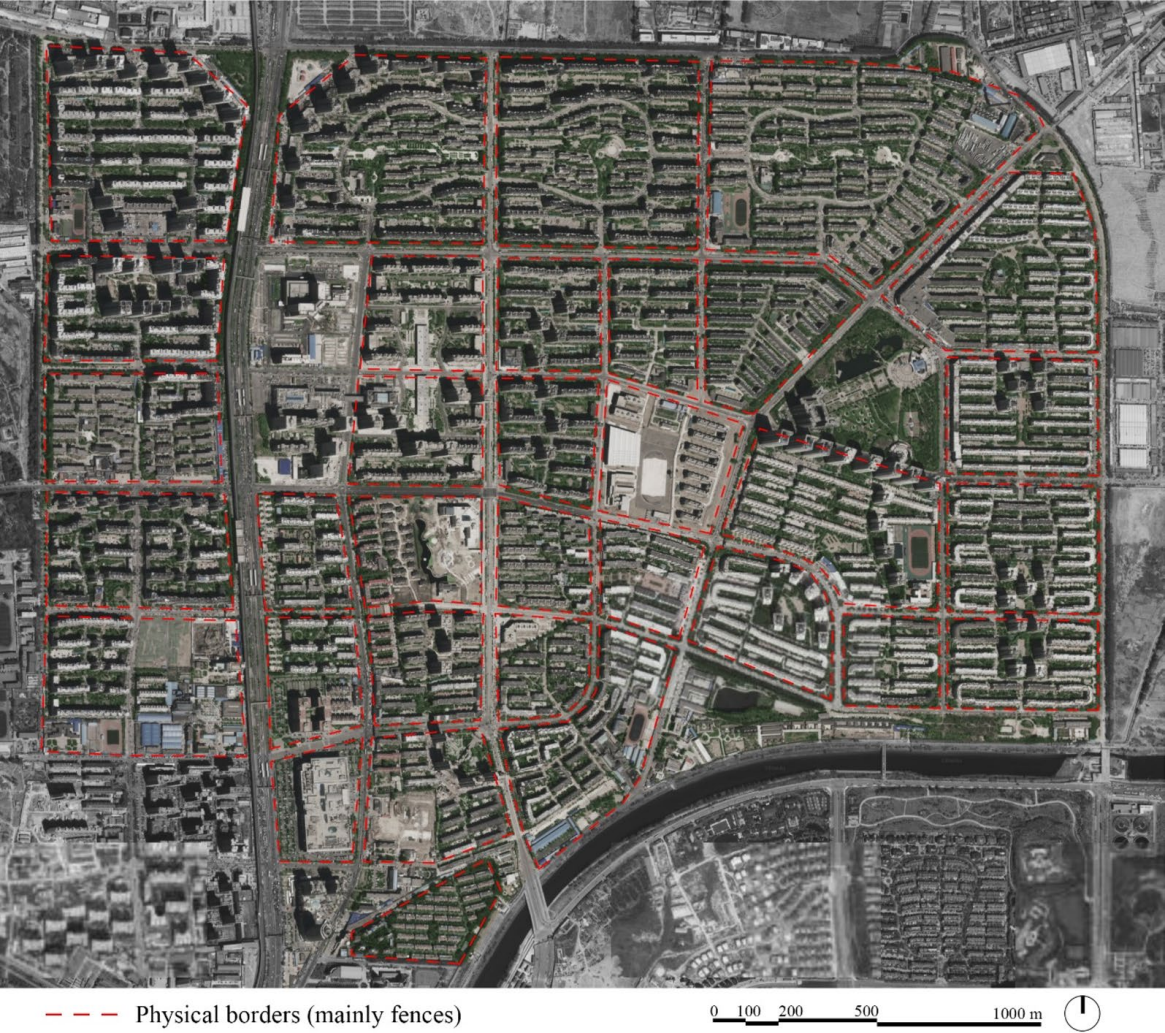
Plan of the residential district of Tiantongyuan, Beijing. Almost all residential blocks are gated and surrounded by fences (Illustrated by the author based on Baidu maps, 2019)

Without any doubt, a real sense of security cannot be obtained by building more walls and gating more neighbourhoods. However, the problems of congestion, pollution and social stratification, cannot be solved simply by dismantling the walls and banning the construction of new gated communities, which were encouraged by the highest authorities themselves making of the gated communities ‘China’s tried and tested investment model for minimum risk residential development’ (Mars and Hornsby 2008). For instance Tiantongyuan, located in northern Beijing and developed in 1998 by Beijing Municipal Government, as one of the first affordable housing districts, to absorb the high request for housing after the collapse of the socialist welfare housing system, is one of the largest residential developments in China with over 400,000 residents, a ‘city within the city’, composed mostly of gated communities separated by wide streets (Fig. 6). However, the problems associated with this area, e.g. traffic congestion, are not the result of the gated community model in se, but of poor planning, functional and spatial organization. In fact, Tiantongyuan was conceived as a ‘sleeping city’, a mono-functional district lacking sufficient public facilities, commercial and cultural services, which generated a strong jobs-housing imbalance forcing a large amount of residents to commute on a daily basis to other far surrounding areas (Meng et al. 2012). Enclosed residential areas physically surrounded by fences or walls and pierced by gates, are a recurrent practice in many Chinese cities, a highly complex and multidimensional urban issue, which solutions cannot be addressed simply through the eradication of its physical borders.

### Gated communities: No ‘one-size-fits-all’ solutions

**Fig. 7:**
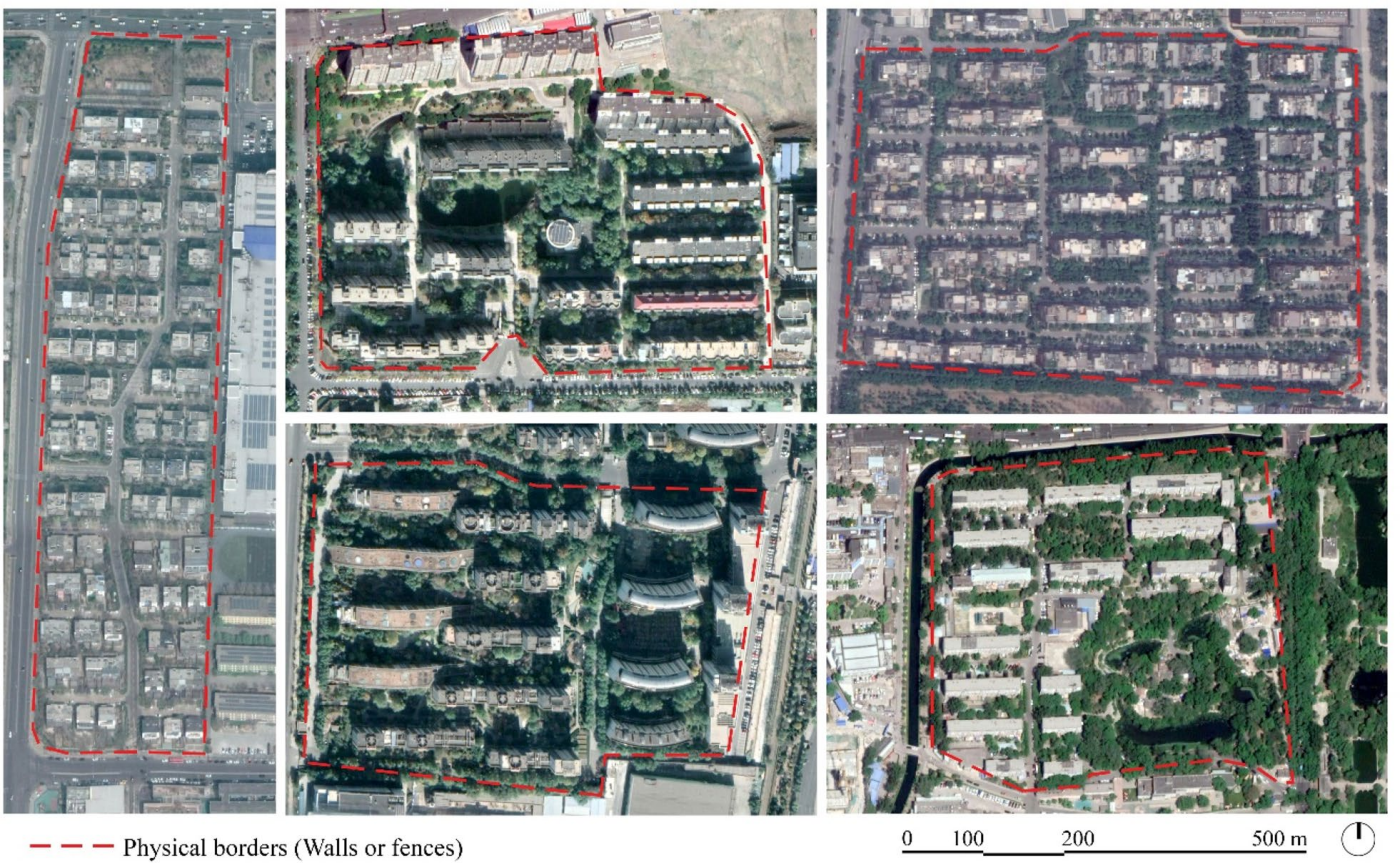
Plans of different gated communities in Haidian District, Beijing. Most of them are large-scale gated superblocks with their own property management systems and structure (Illustrated by the author based on Baidu maps, 2019)

In China with the fast process of urbanization after the economic reforms, the potential of cities to be a device of social organization, more liveable and friendly to citizens, has been overlooked, while much focus was given to their expansion as living containers. The presence on the same territory of different neighbourhoods operating according to their own governance modes and strategies, have resulted in bounded spaces with their specific rules, spatial organization and management. ‘The Chinese government encourages the construction of autonomous residential districts and compounds. Equipped with private security and governed by neighbourhood committees, the enclave saves the state money and relieves it (at least in part) of its responsibilities to administer and maintain order (Mars and Hornsby 2008). Although recognizing the shortcomings of the gated communities such as the large-scale blocks, and the poor connectivity with the surrounding built urban fabric, it is equally important to underline that the gated communities in China have complex and different social, economic, physical and property management systems, which need to be addressed using diversified approaches according to the various situations (Fig. 7).

The idea of opening the gated communities is driven mainly by the urgency to modify the physical environment of Chinese cities, e.g. increasing road connectivity and accessibility, and promoting small-scale residential blocks. However, one of the main tasks before starting any physical transformation is to understand the complex social relationships that govern the physical spaces of the gated communities and how to face the challenge of involving the multiple stakeholders, e.g. street offices, real estate developers, property managers, residents, homeowners’ associations, in the process of transformation, fostering co-creation and co-governance. Moreover, considering the facilitating role gated communities played in the actualization of the confinement measures and social control during the outbreak of COVID-19 in China (Hamama 2020), it is arguable urban enclosure will be revaluated and reinforced further challenging any tentative openness in the near future.

### Rethink the gated communities as multifunctional and vibrant places

In China, the *danwei* system reflects a spatial type of the jobs-housing balance, which has been long in existence and is still correlated to more environmentally friendly transportation mode usages and shorter commutes (Zhou et al. 2014). Spatially, one of the advantages of *danwei* planning pattern is short commutes and mixed uses. On the other hand, it is believed that the decline of *danwei*, accompanied by the process of decentralization, had contributed to the jobs-housing imbalance which increased the commuting times (Wang and Chai 2009). Taking the *danwei* system as a model, the gated communities have the potentials to support less invasive interventions, such as the introduction of new roads to accommodate the increasing vehicular traffic, and contribute instead to the achievement of harmonious cities and qualitative urban spaces taking advantage of the existing physical borders and spatial structure.

**Fig. 8:**
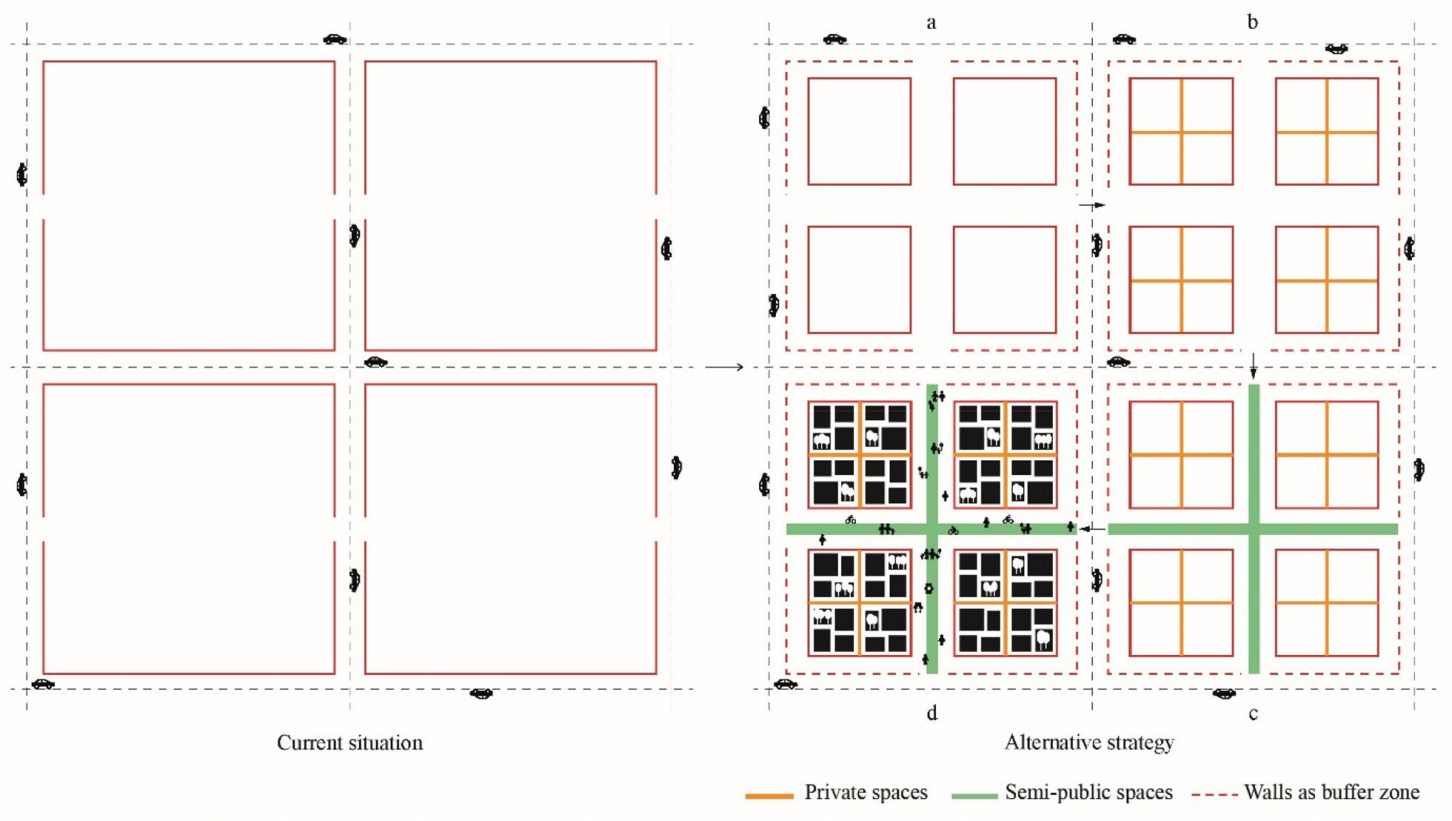
**a** The large-scale blocks could be subdivided into small blocks. **b** Each small block could have its own internal subdivision and private spaces. **c** Streets within the large bocks are meant to be mainly pedestrian with restricted vehicle access. **d** Each large block could support mixed functions. The existing boundaries will function as a ‘buffer zone’ to protect the spaces within the large blocks from noise and pollution, assuring a safe and pedestrian-friendly environment for people (Source: Hamama 2017)

Most gated communities have their own internalized services and amenities, sense of privacy and are pedestrian friendly. Their inner spaces can be transformed into multifunctional and vibrant places supporting shared cultural, leisure, and community services open to a larger number of people, while restricting vehicles access to preserve the sense of privacy and safety. The large-scale gated blocks, could be arranged into small blocks (e.g. 150–200 × 150–200 m on each edge), with their own internal arrangement and private spaces, while increasing accessibility and permeability restricting cars to drive around the existing perimeter of the superblock. The existing walls could be maintained and function as a ‘buffer zone’, a transitional element between the most chaotic and noisy environment dominated by cars, and the less stressful and quiet urban atmosphere, which is intended to be created within the walls, so people can walk and carry their daily activities around without the constant fear of cars and traffic (Fig. 8).

### Public participation and empowerment of the local communities as strategies for the creation of people-oriented cities in China

The transition towards the adoption of an alternative urban form in China cannot be achieved without a bottom-up behavioral change at the scale of the communities, the promotion of an approach focusing more on the human scale, and going beyond the traditional approaches. In a report published by the McKinsey Global Institute (Urban World: Meeting the demographic challenge in cities, 2016), it states that ‘*in an era of pressure on urban populations, a vital ingredient for cities to retain and attract citizens, will depend no more on their economic success measured by their overall GDP, but on the quality of life and the well-being of their citizens*’. In China, due to the dual property right system and the efficiency in managing gated communities rather than open urban blocks, ‘both the property owners and the property management companies prefer the mode of gated communities’ (Zeng et al. 2016). However, the ‘public participation mechanisms and the community management system based on public participation is rather weak, both of which hinder the development of high-quality housing development blocks in China to some extent’ (ibid.). According to Zhang et al. (2018), although the neighborhood as the planning unit was the main focus of Western urban planners, in China the focus at the neighborhood scale received little support from national policy and local government, and was often characterized by ambiguous legislation for the public management of the communities, inadequate public participation and a weak sense of community. Liu et al. (2014) sustain that although top-down and bottom-up strategies had been adopted and tested in China, there is still a significant lack of public participation at the local level. Hence, as the basic unit of Chinese cities’ urban fabric, it is at the scale of the local communities that actions should be taken and policies introduced to sustain and promote healthy and sustainable cities with and for the people, encouraging the residents to take part in the decision processes regarding the urban re-generation of their communities and territories.

## Conclusion

This paper, reflecting on China’s *National New Urbanization Plan* (2014–2020), and its direction to shift from the traditional ‘quantitative’ urban development to ‘qualitative and people-oriented’ approaches, brings to the attention of the readers the important questions of what would be the role of the gated community in the new urban agenda, and how to rethink the gated community as an opportunity. Although, gated communities are the dominant urban form of the Chinese urban fabric, they had been often at the center of hot debates, one in particular was raised by the directive of 2016, calling for banning new gated communities, and encouraging the openness of the existing ones. Through a critical review of the existing studies, this paper shows the complexity and multifaceted aspects associated with the gated communities in the Chinese urban context, as it involves socio-spatial, cultural, political and economic factors, which goes beyond its physical borders.

The complex and diversified social, economic, spatial and political structure of the gated communities cannot be addressed using a single approach. There is a necessity for diversified perspectives, strategies and tools to balance the need for urban renewal, technological innovation and social inclusion. At the physical level, the gated communities with their walls, could support mixed uses offering people quality urban spaces and pedestrian friendly environments. At the social level, they could be transformed into urban laboratories for the promotion of initiatives for the re-imagination of the relationship between the residents and their territory, reinforcement of public participation, and the creation of genuine and culturally rich places that reflects the multifaceted reality of the urban fabric, preserve its local identities and strengthen the sense of belonging.

Although more research is needed to explore accurately the proper mechanisms and strategies to rethink the gated communities, it is nowadays of paramount importance to start looking beyond the alleged negative aspects associated with the physical presence of the gated communities and analyse their opportunities and how the physical and symbolic borders could be exploited and re-imagined for the benefit of the city and the people. The opening up policy addressing the gated communities seems more concerned about improving the physical environment and ease the increasing vehicular traffic in China. However, the urban renewal of gated communities, and Chinese cities in general, should address other vital aspects, e.g. how the upgrading of the physical environment could result in a broader social system improvement, how to encourage the decentralization of power creating the conditions to enable more people to exert their creativity and lead the transformation process at the bottom up and the grassroots community level. Although recognizing the benefits associated with the openness of the gated communities, we argue that the opening up policy remains an unrealistic solution in an urban setting dominated by the typology of the gated community, which will be probably further reinforced after the impeccable role it played during the outbreak of COVID-19 in China.

## Data Availability

Not applicable.

## Footnotes

1 The *danwei,* literally means ‘unit’ and is generally referred to, in the Western literature, as work-unit. The *danwei* represented the main place of employment in the People’s Republic of China prior to the economic reforms, and it was for most urban residents the only means by which they could obtain employment (Wang 2013).

2 According to *China Daily*, an online poll of some 20,000 participants on the news portal Sina.com showed that about 75 percent did not support opening up the gated communities, and roughly 18 percent chose to support it.
